# A descriptive assessment of mitochondrial *COI* genetic diversity and dengue and chikungunya RNA detection in *Aedes aegypti* across eight provinces in Thailand

**DOI:** 10.1186/s13071-026-07539-2

**Published:** 2026-06-23

**Authors:** Padet Siriyasatien, Proawpilart Intayot, Rungfar Boonserm, Rinnara Ampol, Nataya Sutthanont, Nutticha Silakom, Charuai Suwanbamrung, Jonas Schmidt-Chanasit, Atchara Phumee

**Affiliations:** 1 Department of Parasitology, Faculty of Medicine, Chulalongkorn University, Bangkok, Thailand; 2 Center of Excellence in Vector Biology and Vector Borne Diseases, Department of Parasitology, Faculty of Medicine, Chulalongkorn University, Bangkok, Thailand; 3 Pharmaceutical Ingredient and Medical Device Research Division, Research Development and Innovation Department, The Government Pharmaceutical Organization, Bangkok, Thailand; 4 Department of Medical Entomology, Faculty of Tropical Medicine, Mahidol University, Bangkok, Thailand; 5 Program in Bioinformatics and Computational Biology, College of Interdisciplinary and Integrative Studies, Chulalongkorn University, Bangkok, Thailand; 6 Excellent Center for Public Health Research: EC for PHR, Walailak University, Nakhon Si Thammarat, Thailand; 7 Public Health Research Program, School of Public Health, Walailak University, Nakhon Si Thammarat, Thailand; 8 Bernhard-Nocht-Institute for Tropical Medicine, Hamburg, Germany; 9 Faculty of Mathematics, Informatics and Natural Sciences, University of Hamburg, Hamburg, Germany; 10 Department of Medical Technology, School of Allied Health Sciences, Walailak University, Nakhon Si Thammarat, Thailand

**Keywords:** *Aedes aegypti*, Dengue virus, Chikungunya virus, Cytochrome c oxidase subunit I, Genetic diversity, Thailand

## Abstract

**Background:**

*Aedes aegypti* is the principal vector of several medically important arboviruses, including dengue virus (DENV) and chikungunya virus (CHIKV), both of which remain endemic in Thailand. However, studies on arbovirus surveillance and mitochondrial genetic diversity of *Ae. aegypti* populations across broad geographic scales remain limited. This study aimed to assess the detection of DENV and CHIKV and characterize the mitochondrial genetic diversity of *Ae. aegypti* across multiple regions of Thailand.

**Methods:**

A total of 303 adult *Ae. aegypti* mosquitoes collected from eight provinces across five geographic regions of Thailand were screened for DENV and CHIKV RNA. Mitochondrial cytochrome c oxidase subunit I (*COI*) genetic diversity was assessed using haplotype network analysis and analysis of molecular variance (AMOVA).

**Results:**

CHIKV RNA was detected in 51 mosquitoes (16.8%) from four provinces, with the highest detection rates observed in Prachuap Khiri Khan and Bangkok. DENV RNA was not detected in any sample. *COI* analysis revealed moderate to high mitochondrial haplotype diversity and geographic variation in haplotype distribution among populations. Both widely shared and province-specific haplotypes were identified. AMOVA indicated that most genetic variation occurred within populations (93.19%), with significant genetic differentiation among populations (ΦST = 0.068, *P* < 0.001).

**Conclusions:**

CHIKV RNA was detected in *Ae. aegypti* populations from multiple regions of Thailand, whereas DENV RNA was not detected. Mitochondrial *COI* analysis revealed substantial haplotype diversity and geographic variation among populations. These findings contribute to current knowledge of arbovirus occurrence and mosquito genetic diversity in Thailand and provide a foundation for future vector surveillance studies.

**Graphical Abstract:**

**Figure:**
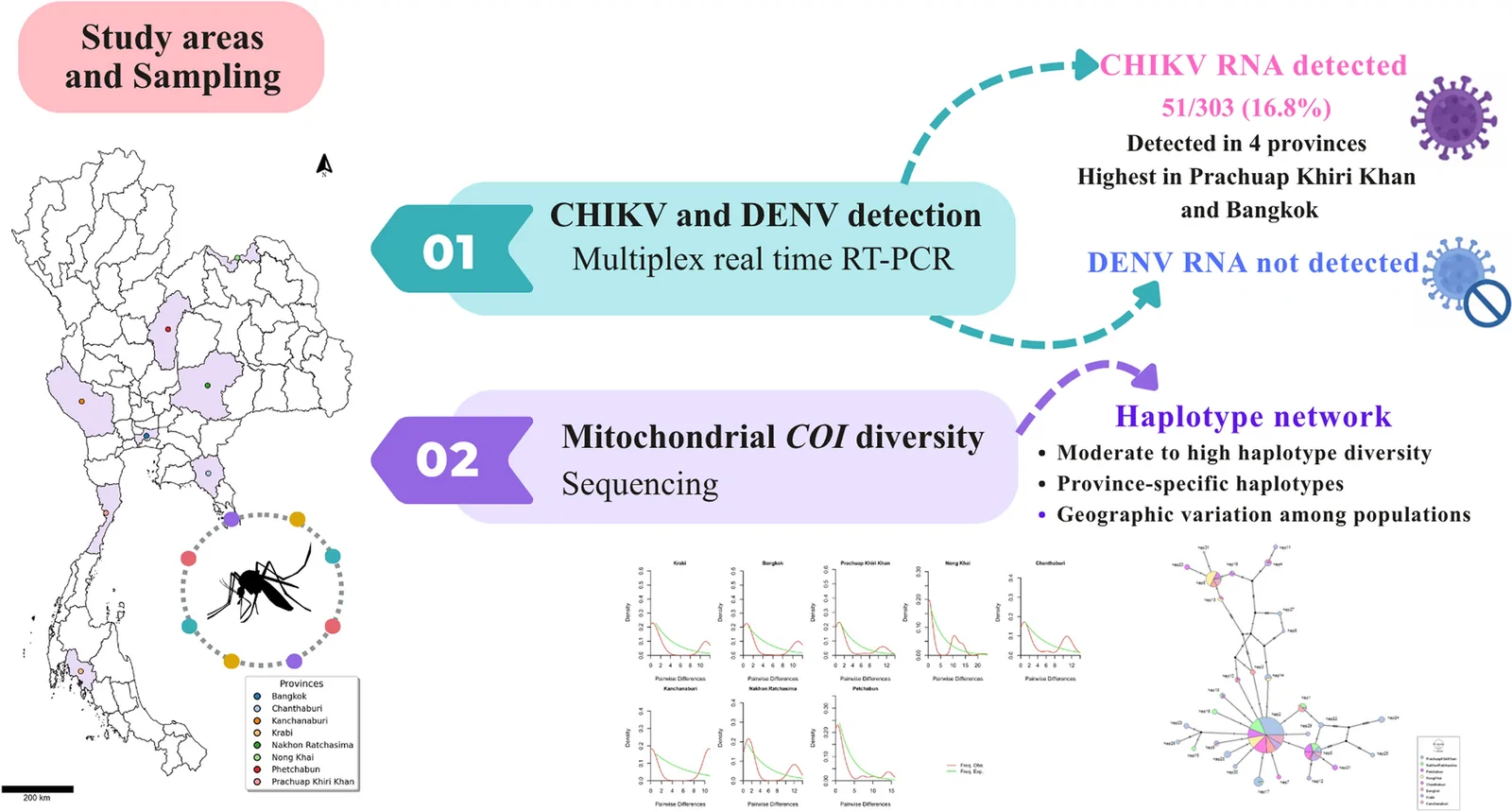

## Background

*Aedes aegypti* is the primary vector responsible for the transmission of several major arboviruses of public health importance, including dengue virus (DENV), Zika virus (ZIKV), chikungunya virus (CHIKV), and yellow fever virus. Its strong ecological association with human habitats, combined with strong anthropophilic and endophilic behaviors, has facilitated widespread transmission and recurrent large-scale epidemics across tropical and subtropical regions worldwide [1]. Genetic and phylogeographic evidence indicates that *Ae. aegypti* originated in Africa from an ancestral sylvatic form (*Ae. aegypti* formosus), which occupied forested environments and fed primarily on non-human vertebrates [2, 3]. Subsequent adaptation to human-associated environments led to the emergence of the domesticated, highly anthropophilic form that now serves as one of the most efficient global vectors of human arboviral diseases [4]. In Southeast Asia and particularly in Thailand, *Ae. aegypti* is firmly established in urban and peri-urban settings, where it contributes substantially to the persistent transmission and co-circulation of multiple arboviruses [5]. Dengue is hyperendemic throughout the country, while CHIKV has re-emerged in recent years, with genetically distinct lineages reported across multiple regions and evidence of co-circulation with DENV and ZIKV [6, 7]. Recent epidemiological studies conducted between 2020 and 2023 confirm sustained transmission and increasing case reports of both dengue and chikungunya in Thailand, underscoring the continuing public health challenge posed by these arboviruses [8, 9]. Beyond human infections, several studies have demonstrated the presence of DENV, CHIKV, and ZIKV in field-collected *Ae. aegypti*, with evidence of vertical transmission detected in both male and female mosquitoes. These findings highlight the potential role of mosquito populations in viral persistence and maintenance independent of active human transmission [10–13]. Collectively, this body of evidence indicates that arboviral diseases continue to exert a substantial burden in Thailand and that mosquito populations contribute to sustaining transmission cycles. Given the limited availability of specific antiviral therapies and broadly effective vaccines, current strategies to reduce arboviral disease burden rely largely on vector control interventions and early clinical management. From a molecular entomology perspective, understanding mosquito population history, genetic structure, and spatial patterns of genetic diversity can support vector research in endemic settings. Mitochondrial DNA markers, particularly the *COI* gene, are widely used for species identification and haplotype characterization in mosquito vectors, although their resolution for population-level inference is limited to maternal lineages. Despite the epidemiological importance of *Ae. aegypti* in Thailand, studies examining mitochondrial genetic diversity across multiple geographic regions remain limited [14, 15]. This study therefore aimed to characterize the spatial patterns of mitochondrial *COI* genetic diversity in *Ae. aegypti* across eight provinces representing different regions of Thailand. In parallel, field-collected mosquitoes were screened for DENV and CHIKV RNA to document viral detection in natural vector populations.

## Methods

### Mosquito collection

Mosquito samples were collected from eight provinces representing five geographical regions of Thailand: Northeastern (Nakhon Ratchasima, NMA; Nong Khai, NK), Central (Bangkok, BKK; Phetchabun, PNB), Western (Prachuap Khiri Khan, PC; Kanchanaburi, KRI), Eastern (Chanthaburi, C), and Southern (Krabi, KB). Sampling locations were recorded at the province level, and precise geographic coordinates were not systematically collected. Indoor and outdoor collections were conducted within the same village areas, although distances between specific sampling points were not measured. Mosquito collections were conducted between November 2020 and April 2021, spanning the transition from the rainy to the dry season in Thailand. Sampling was  performed in villages with previous reports of CHIKV and DENV outbreaks. Adult mosquitoes were collected using mosquito aspirators and insect catching nets. In addition, third- and fourth-instar larvae and pupae were sampled from approximately 50 water-holding containers per site, including outdoor water jars, old tires, uncovered jars, indoor cisterns, and coconut shells located in and around households. To minimize potential sampling bias from closely related individuals, immature stages were collected from multiple containers across each site rather than repeatedly sampling from the same container. However, the number of individuals collected per container was not systematically recorded, and the possibility of sampling siblings cannot be excluded. Sampling was distributed across collection areas to capture a range of microhabitats, although precise spatial coordinates and distances between collection points were not recorded. Larvae and pupae were reared to adults under laboratory conditions and morphologically identified to species using standard taxonomic keys [16]. Both male and female adult mosquitoes were included in the analyses. All adult mosquito specimens were preserved in liquid nitrogen and transported to the laboratory for further processing.

### Nucleic acid extraction of mosquitoes

Nucleic acid extraction was performed on individual *Ae. aegypti* samples without pooling. Each mosquito was placed in a 1.5 ml microcentrifuge tube containing 200 µl of 1 × phosphate-buffered saline (PBS), homogenized thoroughly, and centrifuged at 13,000 × g for 10 min. From the clarified supernatant, 200 µl was loaded into the Magtration magLEAD 12gC system (Precision System Science, Japan) and processed with MagDEA Dx reagents according to the manufacturer’s protocol. Total nucleic acids (RNA and DNA) were extracted, eluted in 50 µl of elution buffer, and stored at − 80 °C until further analysis.

### *COI* gene amplification in mosquito samples

A fragment of the *COI* gene was amplified using the forward primer C1-J-1718-F (5ʹ-GGAGGATTTGGAAATTGATTAGTTCC-3ʹ) and the reverse primer C1-N-2191-R (5ʹ-CCCGGTAAAATTAAAATATAAACTTC-3ʹ), as described by Simon et al. [17]. PCR amplification was performed in a 25 µl reaction mixture containing 2 µl of DNA template, 2.5 µl of 10 × PCR buffer, 1.25 µl of 25 mM MgCl_2_, 1.25 µl of 10 µM dNTPs, 0.85 µl of each primer (10 µM), 0.2 µl of *Taq* DNA polymerase (5 U/µl; Thermo Scientific, USA), and 16.1 µl of sterile nuclease-free water. The thermocycling conditions consisted of an initial denaturation at 95 °C for 5 min; followed by 35 cycles of denaturation at 94 °C for 30 s, annealing at 51 °C for 30 s, and extension at 72 °C for 45 s; with a final extension at 72 °C for 10 min. For gel electrophoresis, 10 µl of each PCR product was mixed with 1 µl of fluorescent loading dye (Boca Scientific, USA) and loaded onto a 1.5% agarose gel. DNA bands were visualized under UV illumination using a Gel Doc EQ System (Bio-Rad, USA). The presence of the expected 524 bp amplicon was confirmed by comparison with a 100 bp DNA ladder (Thermo Scientific, USA).

### Screening of DENV and CHIKV RNAs

All samples were screened for DENV and CHIKV RNA using a one-step multiplex real-time RT-PCR assay. The reactions were performed with the abTES DEN 5 qPCR I Kit (AIT Biotech Pte. Ltd., Singapore), a multiplex real-time RT-PCR assay capable of simultaneously detecting and differentiating all four DENV serotypes (DENV-1 to DENV-4) and CHIKV, following the manufacturer’s instructions. Each reaction was carried out in a total volume of 25 µl, comprising 12.5 µl of 2 × RT-PCR reaction mix, 0.5 µl of RT/Taq enzyme mix, 1.5 µl of primer/probe mix, 0.1 µl of PCR enhancer, 5.4 µl of nuclease-free water, and 5.0 µl of template. Amplification was conducted on a CFX96 Real-Time PCR Detection System (Bio-Rad Laboratories, USA) under the following thermal cycling conditions: reverse transcription at 53 °C for 10 min, initial denaturation at 95 °C for 2.5 min, followed by 41 cycles of denaturation at 95 °C for 17 s, annealing at 59 °C for 31 s, and extension at 68 °C for 32 s. A final extension step was performed at 68 °C for 7 min, and reactions were held at 4 °C until analysis. Samples were considered positive when amplification curves crossed the threshold within Ct < 38, whereas samples with Ct ≥ 38 or no amplification were considered negative, in accordance with the manufacturer’s recommended cutoff values.

### Mitochondrial *COI* sequence analysis and population genetic inference

Raw chromatograms were inspected and edited using BioEdit (v7.7.1.0). Low-quality bases were trimmed based on Phred quality scores (Q ≥ 20), and ambiguous base calls were manually checked and corrected. Forward and reverse sequences were assembled into consensus sequences and verified to ensure base-calling accuracy. After quality control, high-quality *COI* sequences of 524 bp were obtained from 303 mosquito samples. All sequences were aligned with the *COI* gene sequences of all mosquitoes using MAFFT version 7 [18]. The resulting alignments were inspected to ensure correct reading frame for this protein-coding region. Identical sequences were collapsed into unique haplotypes, and a haplotype table was generated summarizing haplotype frequencies across populations and identifying private haplotypes. Haplotype indices, including the number of haplotypes (H), haplotype diversity (Hd), nucleotide diversity (π), and the average number of nucleotide differences (k), were calculated using the pegas package in R [19]. The genealogical relationships among haplotypes were visualized using a TCS haplotype network constructed and plotted in PopART v1.7. [20]. Mismatch distribution analyses were performed to examine the distribution of pairwise nucleotide differences. Neutrality was assessed using Tajima’s D, with statistical significance evaluated using analytical P-values assuming a normal distribution with mean zero. Population genetic structure was quantified using analysis of molecular variance (AMOVA) to partition genetic variation within and among populations, and the significance of variance components and ΦST values was tested using permutation procedures. All analyses were conducted using the R package pegas.

## Results

### Detection of CHIKV, DENV, and mosquito *COI* sequences

**Fig. 1:**
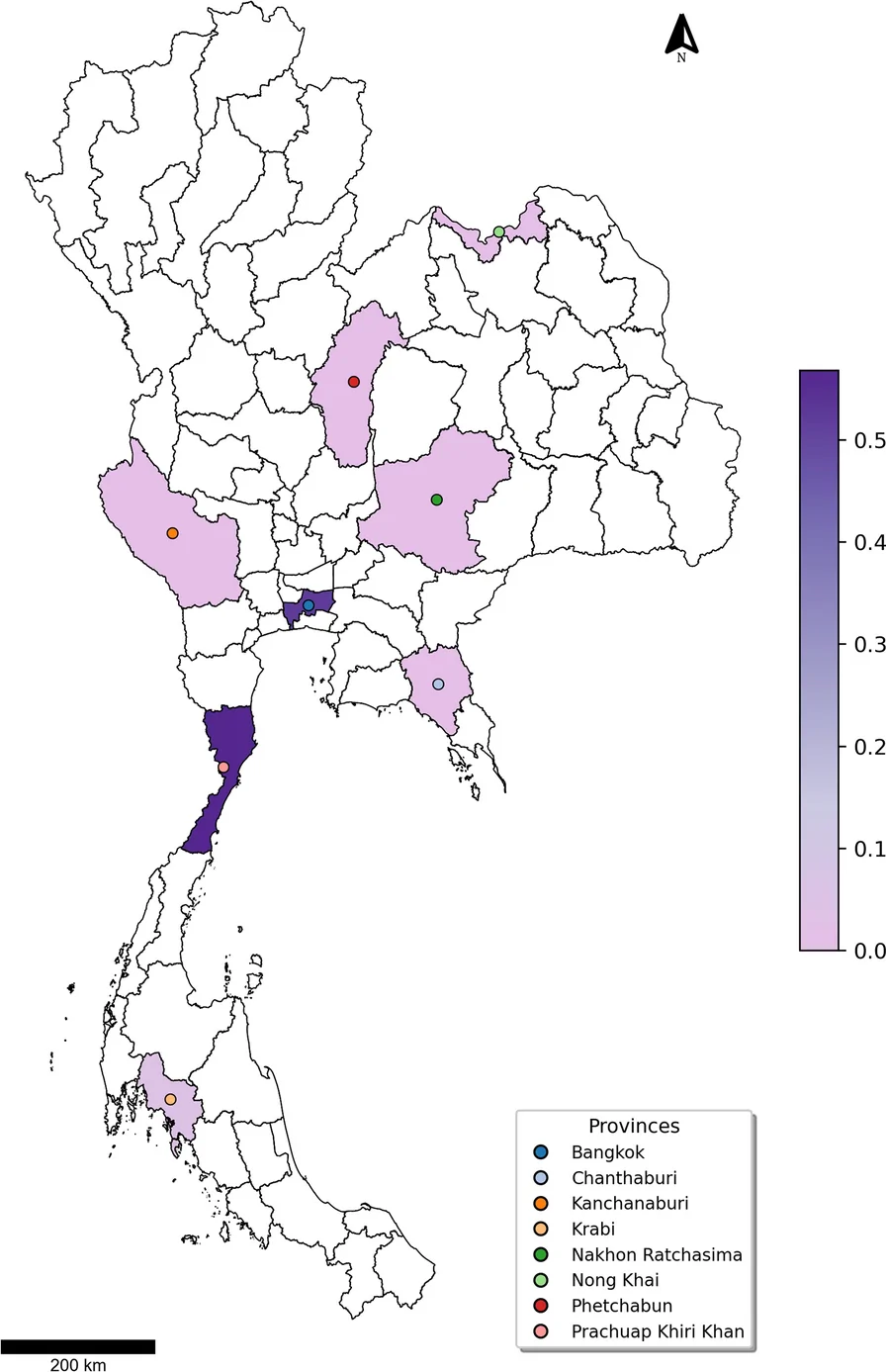
Spatial distribution of CHIKV detection rates across eight sampling provinces in Thailand. Detection rates are represented using a color gradient from low (light purple) to high (dark purple). The provinces included are Bangkok, Chanthaburi, Kanchanaburi, Krabi, Nakhon Ratchasima, Nong Khai, Phetchabun, and Prachuap Khiri Khan. Colored point markers indicate the specific sampling locations within each province

A total of 303 *Ae. aegypti* mosquitoes collected from eight provinces representing five geographic regions of Thailand were screened for CHIKV and DENV RNA. Overall, CHIKV RNA was detected in 51 samples, corresponding to a detection rate of 16.8%. Among these, 37 of 204 females (18.1%) and 14 of 99 males (14.1%) tested positive. CHIKV-positive mosquitoes were identified in four provinces. The highest detection rates were observed in Prachuap Khiri Khan Province (Western region), where CHIKV was detected in 25 of 44 females (56.8%) and 8 of 47 males (17.0%). Bangkok (Central region) also showed relatively high detection rates, with CHIKV detected in 11 of 21 females (52.4%) and 3 of 7 males (42.9%). Lower detection rates were observed in Nong Khai Province, where CHIKV was detected exclusively in males (3/16, 18.8%), and in Krabi Province, where CHIKV was detected in 1 of 25 females (4.0%). No CHIKV-positive mosquitoes were detected in samples from Nakhon Ratchasima, Phetchabun, Kanchanaburi, or Chanthaburi provinces (Table 1, Fig. 1). In contrast, RNA corresponding to all four dengue virus serotypes (DENV-1 to DENV-4) was not detected in any of the mosquito samples examined. *COI* sequences were successfully obtained from all 303 samples and used for subsequent population genetic analyses. All *COI* sequences have been deposited in the NCBI GenBank database under accession numbers PX706302-PX706604.

**Table 1:** Sample collection and chikungunya virus (CHIKV) detection in *Ae. aegypti* mosquitoes from eight provinces in Thailand

Region	Provinces	CHIKV-positive		*COI* sequences (n)
		Female n/total (%)	Male n/total (%)	
Northeastern	Nakhon Ratchasima	0/31 (0.0)	0/5 (0.0)	36
	Nong Khai	0/17 (0.0)	3/16 (18.8)	33
Central	Bangkok	11/21 (52.4)	3/7 (42.9)	28
	Phetchabun	0/26 (0.0)	0/9 (0.0)	35
Western	Prachuap Khiri Khan	25/44 (56.8)	8/47 (17.0)	91
	Kanchanaburi	0/19 (0.0)	0/5 (0.0)	24
Eastern	Chanthaburi	0/21 (0.0)	0/8 (0.0)	29
Southern	Krabi	1/25 (4.0)	0/2 (0.0)	27
Total		37/204 (18.1)	14/99 (14.1)	303

*CHIKV* chikungunya virus, *COI* cytochrome c oxidase subunit I. Values are presented as number of CHIKV-positive mosquitoes per total tested, with detection rates expressed as percentages

### Geographic patterns of mitochondrial *COI* genetic diversity

**Fig. 2:**
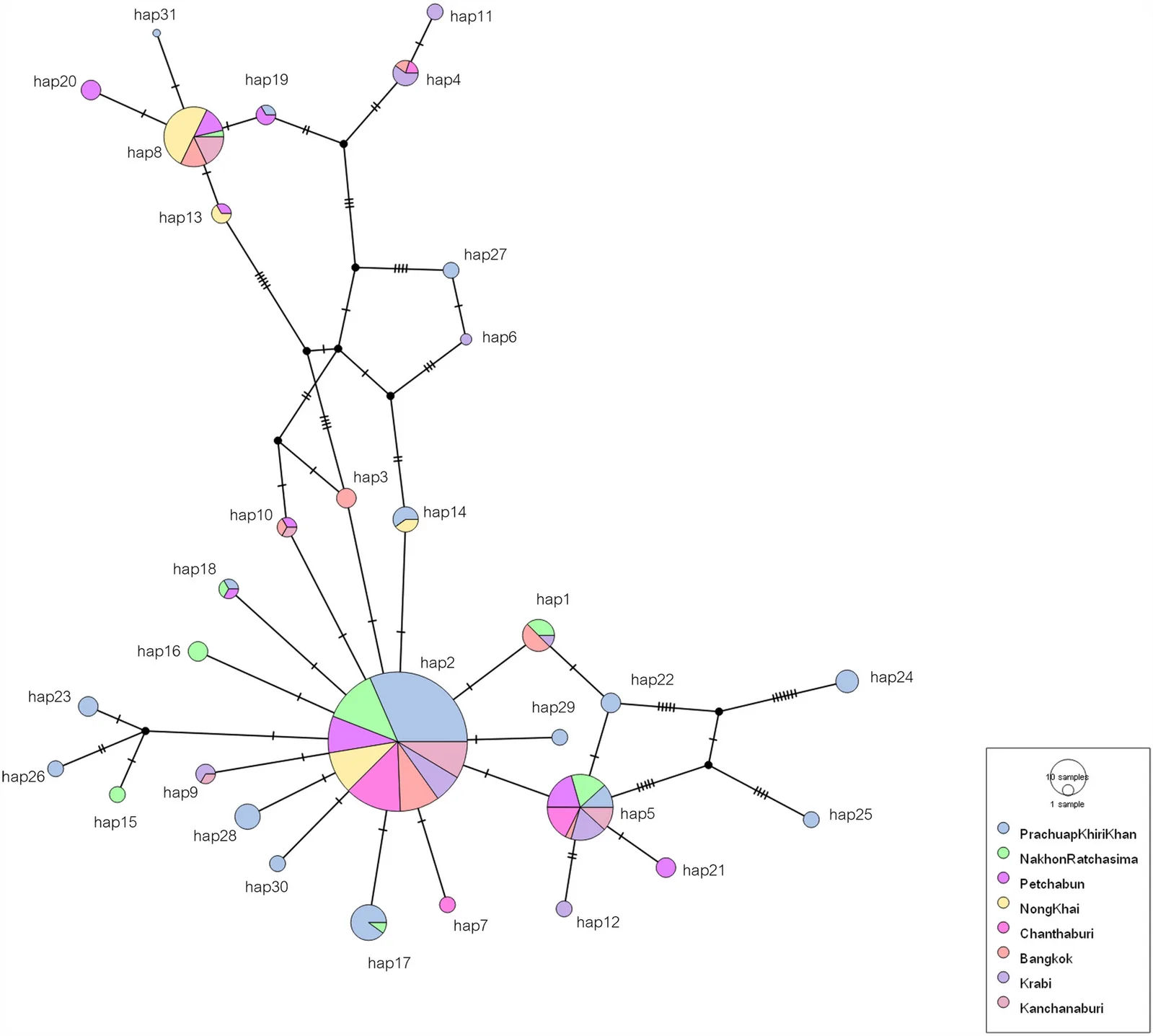
TCS haplotype network of *Ae. aegypti COI* sequences from eight provinces in Thailand. Each circle represents a unique haplotype, with circle size proportional to the number of individuals carrying that haplotype. Pie-chart colors indicate the provincial origin of samples. Hash marks on connecting lines represent mutational steps between haplotypes. The network illustrates both shared haplotypes across provinces and province-specific variants, reflecting variation in mitochondrial *COI* haplotype composition among sampling locations

A total of 303 *COI* sequences obtained from *Ae. aegypti* across eight provinces of Thailand revealed geographic variation in haplotype composition and genetic diversity. The TCS haplotype network identified both widely shared haplotypes across multiple provinces and province-specific haplotypes (Fig. 2). Across sampling sites, haplotype richness (H = 5–15) and haplotype diversity (Hd = 0.60837–0.81177) varied among populations. The highest haplotype diversity was observed in Phetchabun and Krabi, whereas Chanthaburi exhibited the lowest diversity, consistent with its limited number of closely related haplotype in the network. Nucleotide diversity (π) also varied among provinces, ranging from 0.00292 in Nakhon Ratchasima to 0.01101 in Nong Khai. Prachuap Khiri Khan, which had the largest sample size and highest number of haplotypes, showed moderate Hd and low π, reflecting the presence of multiple closely related haplotype (Table 2).

**Table 2:** Summary of mitochondrial *COI* haplotype diversity of *Ae. aegypti* populations across eight provinces in Thailand

Province	Haplotype (No. sequence)	N	S	H	Hd	π	k
Bangkok	Hap2(15), Hap1(4), Hap3(3), Hap8(3), Hap10(1), Hap4(1), Hap5(1)	28	15	7	0.69048	0.00629	3.29629
Chanthaburi	Hap2(17), Hap5(7), Hap4(2), Hap7(2), Hap6(1)	29	15	5	0.60837	0.00425	2.22660
Kanchanaburi	Hap2(12), Hap8(6), Hap5(4), Hap10(1), Hap9(1)	24	14	5	0.68478	0.00909	4.76087
Krabi	Hap2(10), Hap5(6), Hap4(4), Hap11(2), Hap12(2), Hap1(1), Hap6(1), Hap9(1)	27	19	8	0.80627	0.00943	4.94017
Nakhon Ratchasima	Hap2(19), Hap5(6), Hap1(3), Hap16(3), Hap15(2), Hap17(1), Hap18(1), Hap8(1)	36	17	8	0.69365	0.00292	1.53175
Nong Khai	Hap2(15), Hap8(12), Hap13(2), Hap14(2), Hap31(2)	33	12	5	0.67046	0.01101	5.76894
Petchabun	Hap2(13), Hap5(7), Hap8(4), Hap20(3), Hap21(3), Hap19(2), Hap10(1), Hap13(1), Hap18(1)	35	16	9	0.81177	0.01029	5.39496
Prachuap Khiri Khan	Hap2(48), Hap17(8), Hap28(5), Hap5(5), Hap24(4), Hap14(3), Hap22(3), Hap23(3), Hap25(2), Hap26(2), Hap27(2), Hap29(2), Hap30(2), Hap18(1), Hap19(1)	91	34	15	0.70794	0.00568	2.97631

*N* number of sequences, *S* number of segregating sites, *H* number of haplotypes, *Hd* haplotype diversity, *π* nucleotide diversity per site, *k* average number of nucleotide differences

### Demographic history based on mismatch distribution and neutrality tests

**Fig. 3:**
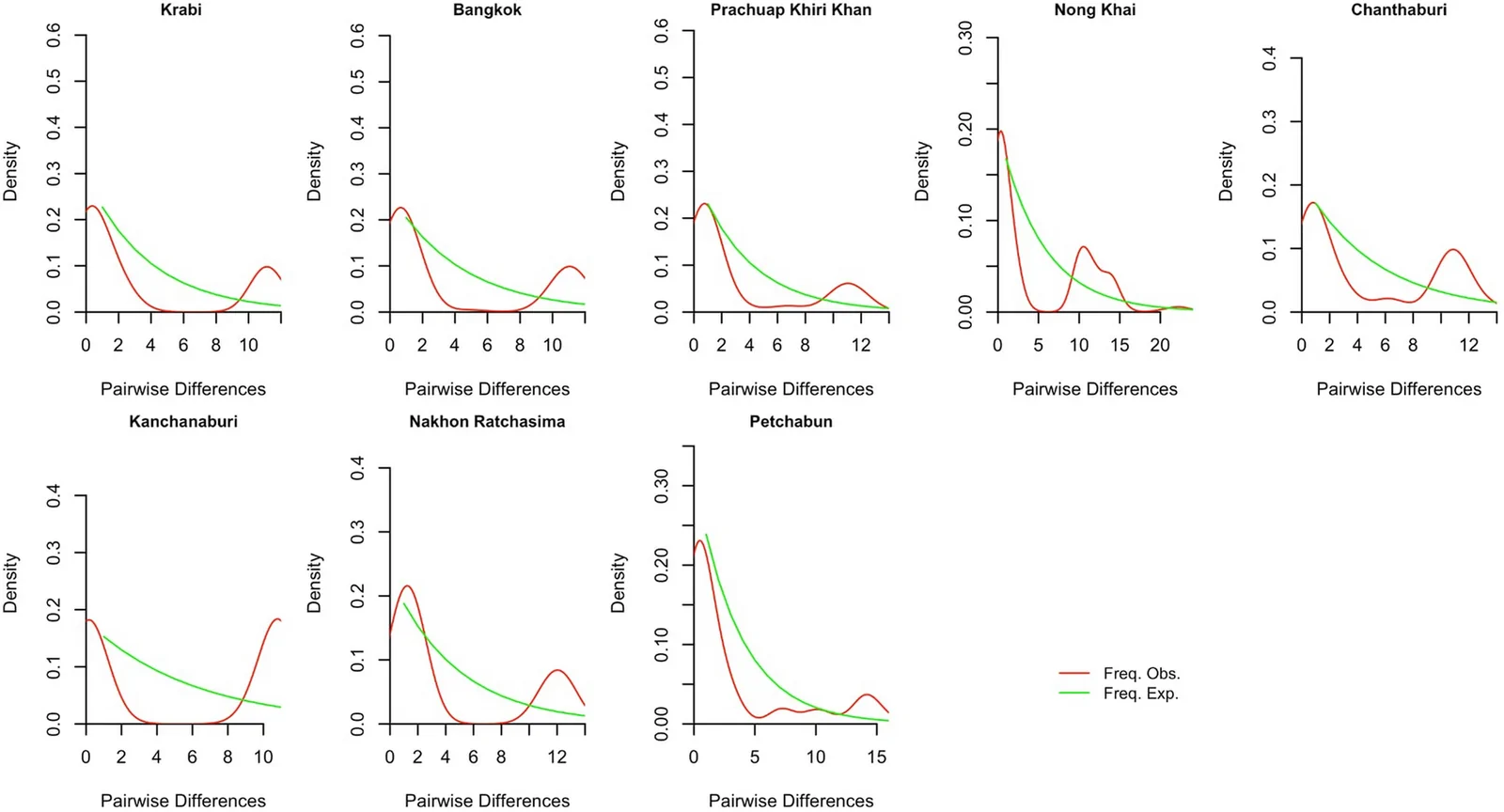
Mismatch distribution plots based on *COI* sequences from eight sampling locations in Thailand: Krabi, Bangkok, Prachuap Khiri Khan, Nong Khai, Chanthaburi, Kanchanaburi, Nakhon Ratchasima, and Phetchabun. Red curves represent the observed frequency distribution of pairwise differences, while green curves indicate the expected distribution under a sudden expansion model

Mismatch distribution analyses revealed variation in the distribution of pairwise nucleotide differences among *Ae. aegypti* populations across Thailand. Several populations, including Krabi, Bangkok, Chanthaburi, Nakhon Ratchasima, and Kanchanaburi showed irregular or multimodal distributions. In contrast, populations from Nong Khai, Phetchabun, and Prachuap Khiri Khan exhibited more unimodal distributions, although minor deviations were observed. Neutrality tests based on Tajima’s D generally supported patterns observed in the mismatch distribution analyses. Significant departures from neutrality were detected in the Nong Khai (Tajima’s D = 3.04228, *P* < 0.05) and Nakhon Ratchasima (Tajima’s D = −2.06971, *P* < 0.05), while all other populations showed non-significant values (Fig. 3). Overall, these results indicate geographic variation in mitochondrial genetic patterns among *Ae. aegypti* populations across Thailand (Table 3).

**Table 3:** Neutrality test of *Ae. aegypti* populations based on the mitochondrial *COI* gene, calculated using the R package pegas

Population	N	Nha	Hd	Pi	Tajima_D	Pb
Bangkok	28	7	0.69048	0.00629	− 0.49487	0.62069
Chanthaburi	29	5	0.60837	0.00424	− 1.41453	0.15720
Kanchanaburi	24	5	0.68478	0.00908	0.94549	0.34440
Krabi	27	8	0.80628	0.00943	0.00773	0.99383
Nong Khai	33	5	0.67045	0.01101	3.04228	0.00235*
Nakhon Ratchasima	36	8	0.69365	0.00292	− 2.06971	0.03848*
Petchabun	35	9	0.81176	0.01029	1.28078	0.20027
Prachuap Khiri Khan	91	15	0.70793	0.00568	− 1.72832	0.08393

*P < 0.05, *N* number of sequences, *Nha* number of haplotypes, *Pb* P-value of Tajima’s D assuming a normal distribution with mean zero

### Analysis of molecular variance (AMOVA)

Analysis of molecular variance (AMOVA) based on mitochondrial *COI* sequences showed that most genetic variation in *Ae. aegypti* was partitioned within populations (93.19%), while a smaller but statistically significant proportion occurred among populations (6.81%; ΦST = 0.068, P < 0.001). These results indicate low but statistically significant differentiation among populations at the mitochondrial *COI* level across the sampled provinces (Table 4).

**Table 4:** Analysis of molecular variance (AMOVA) based on *COI* sequences of *Ae. aegypti*

Molecular Marker	Mosquito	Source of variation	df	Sum of squares	Variance components	Percentage of variation (%)	Fst (ΦST)
*COI*	*Ae. aegypti*	Among populations	7	0.00194	5.55E-06	6.81	0.068***
		Within populations	295	0.02241	7.6E-05	93.19	
		Total	302	0.02435	8.15E-05	100	

ΦST represents the analogue of FST derived from AMOVA of mitochondrial *COI* sequences

^***^P < 0.001, based on 1000 permutations

## Discussion

Arboviral infections, particularly those caused by DENV, CHIKV, and ZIKV, remain a major public health concern in Thailand, where *Aedes* mosquitoes are widely distributed across urban, peri-urban, and rural environments [21, 22]. Previous entomo-virological and molecular surveillance studies have documented continued circulation of CHIKV and other arboviruses in mosquito populations in the region [9, 12, 23]. In the present study, the detection of CHIKV RNA in field-collected *Ae. aegypti* provides evidence of ongoing viral circulation within mosquito populations in Thailand. The identification of CHIKV in both female and male mosquitoes, together with the absence of detectable DENV RNA in all samples, suggests variability in arbovirus detection across locations and time. These findings are consistent with previous reports indicating that CHIKV can persist within mosquito populations independently of concurrent dengue transmission, potentially through mechanisms such as vertical transmission or localized amplification cycles [24–26]. Notably, the high CHIKV detection rates observed in Prachuap Khiri Khan and Bangkok may reflect geographic variation in CHIKV circulation among the sampled *Ae. aegypti* populations, which could be associated with differences in vector density, human movement patterns, or environmental conditions favorable for viral amplification. This spatial heterogeneity highlights the value of continued entomological surveillance for monitoring arbovirus circulation across different geographic locations and supporting the early detection of emerging transmission risks [27].

Genetic diversity and population structure provide important insights into population dynamics, dispersal, and adaptive potential of mosquito vectors, which may ultimately influence pathogen transmission and epidemiological spread [28]. Mitochondrial markers such as the *COI* gene are widely used to assess genetic diversity because of their maternal inheritance and moderate evolutionary rate [29, 30]. In this study, mitochondrial *COI* analysis revealed high haplotype richness (H = 5–15) and moderate to high haplotype diversity (Hd = 0.60837–0.81177), coupled with low nucleotide diversity across Thai *Ae. aegypti* populations. Distinct haplotype compositions among provinces further suggest spatial variation in mitochondrial genetic diversity. Similar geographic patterns have been reported previously and have been attributed to both natural mosquito dispersal and human-mediated movement [28, 31]. According to the framework of Grant and Bowen (1998), the *Ae. aegypti* populations examined in this study fall within the “high Hd-low π” category, a pattern characterized by numerous closely related haplotypes differing by only a few nucleotide substitutions [32] . Previous studies have suggested that this genetic signature is often associated with demographic expansion or rapid population growth following historical bottlenecks [32]. Comparable patterns have been reported in *Ae. aegypti* populations from Benin [33], western Kenya [34], and Penang, Malaysia [30], where high haplotype diversity and low nucleotide diversity were interpreted as evidence consistent with population expansion. Song et al. (2024) further suggested that high haplotype richness and diversity may contribute to increased adaptive potential under changing environmental conditions [35]. Conversely, studies from China reported lower mitochondrial diversity in *Ae. aegypti* compared with the more widely distributed *Ae. albopictus*, highlighting the influence of invasion history, geographic distribution, and ecological adaptation on species-specific genetic patterns [36]. To further characterize genetic variation, mismatch distribution analyses, Tajima’s D neutrality tests, and AMOVA were performed. Together, these analyses revealed high within-population mitochondrial diversity, reflected by elevated haplotype diversity, alongside low but statistically significant differentiation among populations. Comparable population genetic patterns have been reported in *Ae. aegypti* populations from western Kenya and West Africa, where high haplotype diversity, weak spatial structure, and signals consistent with localized demographic expansion were observed using comparable analytical approaches [33, 34]. Stronger genetic structuring has also been documented in settings characterized by pronounced ecological barriers or restricted human movement, such as island systems or geographically isolated rural regions, where demographic histories tend to be more homogeneous within regions [36, 37]. Overall, these findings indicate substantial mitochondrial genetic variation within Thai *Ae. aegypti* populations and broadly align with patterns reported from other geographic regions [32, 38, 33]. However, no definitive conclusions can be drawn regarding underlying demographic processes such as population expansion, gene flow, or demographic stability because this study relied on a single mitochondrial marker and a limited sampling design. These findings highlight the importance of considering regional variation when interpreting genetic data from mosquito populations. Nevertheless, several limitations should be acknowledged. First, reliance on a single mitochondrial *COI* marker limits inference to maternal lineages and does not reflect nuclear genomic structure or fine-scale gene flow. Second, the sampling strategy, which involved larval collections from water containers, may include closely related individuals, potentially biasing haplotype frequency estimates. Third, the relatively small sample size and coarse spatial resolution at the provincial level limit the detection of fine-scale population patterns. In addition, sampling during the peak rainy season was limited due to logistical constraints related to field accessibility and site conditions, which may have influenced virus detection rates and limited the assessment of seasonal patterns. Finally, the absence of viral genomic data and temporal resolution restricts interpretation of transmission dynamics. Therefore, these findings should be interpreted as descriptive baseline observations rather than representative estimates of population-level patterns or virus prevalence, given the limited sample size, uneven sampling across provinces, and restricted spatiotemporal coverage of the study.

## Conclusions

CHIKV RNA was detected in *Ae. aegypti* from four provinces in Thailand, with no DENV RNA identified in the sampled populations. Mitochondrial *COI*-based analysis revealed moderate to high haplotype diversity and geographic variation in haplotype distribution, with most genetic variation occurring within populations and low but significant differentiation among populations. These findings provide baseline information on CHIKV occurrence and mitochondrial *COI* genetic diversity in *Ae. aegypti* from Thailand. Further studies incorporating larger sample sizes, finer-scale spatial sampling, nuclear genetic markers, and viral genomic data are needed to better characterize mosquito population structure and explore its potential association with arbovirus circulation.

## Data Availability

All data generated or analyzed during this study are included in this published article. Newly generated sequences have been deposited in the GenBank database under accession numbers PX706302-PX706604.

## Abbreviations

*Ae.*: *Aedes*
AMOVA: Analysis of molecular variance
CHIKV: Chikungunya virus
*COI*: Cytochrome c oxidase subunit I
DENV: Dengue virus
DNA: Deoxyribonucleic acid
H: Number of haplotypes
Hd: Haplotype diversity
k: Average number of nucleotide differences
NCBI: National Center for Biotechnology Information
RNA: Ribonucleic acid
ZIKV: Zika virus
π: Nucleotide diversity
